# Individual tree crown delineation and tree species classification with hyperspectral and LiDAR data

**DOI:** 10.7717/peerj.6227

**Published:** 2019-01-11

**Authors:** Michele Dalponte, Lorenzo Frizzera, Damiano Gianelle

**Affiliations:** Department of Sustainable Agro-Ecosystems and Bioresources, Research and Innovation Centre, Fondazione Edmund Mach, San Michele all’Adige, Trento, Italia

**Keywords:** Ecology, Forestry, Remote sensing, Crown delineation, Image classification

## Abstract

An international data science challenge, called National Ecological Observatory Network—National Institute of Standards and Technology data science evaluation, was set up in autumn 2017 with the goal to improve the use of remote sensing data in ecological applications. The competition was divided into three tasks: (1) individual tree crown (ITC) delineation, for identifying the location and size of individual trees; (2) alignment between field surveyed trees and ITCs delineated on remote sensing data; and (3) tree species classification. In this paper, the methods and results of team Fondazione Edmund Mach (FEM) are presented. The ITC delineation (Task 1 of the challenge) was done using a region growing method applied to a near-infrared band of the hyperspectral images. The optimization of the parameters of the delineation algorithm was done in a supervised way on the basis of the Jaccard score using the training set provided by the organizers. The alignment (Task 2) between the delineated ITCs and the field surveyed trees was done using the Euclidean distance among the position, the height, and the crown radius of the ITCs and the field surveyed trees. The classification (Task 3) was performed using a support vector machine classifier applied to a selection of the hyperspectral bands and the canopy height model. The selection of the bands was done using the sequential forward floating selection method and the Jeffries Matusita distance. The results of the three tasks were very promising: team FEM ranked first in the data science competition in Task 1 and 2, and second in Task 3. The Jaccard score of the delineated crowns was 0.3402, and the results showed that the proposed approach delineated both small and large crowns. The alignment was correctly done for all the test samples. The classification results were good (overall accuracy of 88.1%, kappa accuracy of 75.7%, and mean class accuracy of 61.5%), although the accuracy was biased toward the most represented species.

## Introduction

The National Ecological Observatory Network—National Institute of Standards and Technology (NEON-NIST) data science evaluation challenge (Marconi et al., 2018) was a competition with the goal to challenge scientists on three tasks that are central in converting remote sensing images into vegetation diversity and structure information traditionally collected by ecologists: (1) individual tree crown (ITC) delineation, for identifying the location and size of individual trees; (2) alignment between field surveyed trees and ITCs delineated on remote sensing data; and (3) tree species classification.

Individual tree crowns delineation is an automatic procedure that allows the detection of the position, the size, and the shape of ITCs in a remote sensing scene. This procedure is extremely useful in ecological studies as it allows researchers to analyse a forest in its primary element, the tree. Indeed, from the delineated ITCs it is possible to have estimates of the height, the diameter at breast height, the biomass, and the species of a tree (Coomes et al., 2017; Dalponte et al., 2018). At the current status of the research, the main drawback of ITCs delineation algorithms is that they rarely detect all the trees in a scene, as very small trees or trees that are not in a dominant canopy position are normally not visible in a remote sensing image. There is a large amount of literature about ITC delineation (Popescu, Wynne & Nelson, 2003; Lee & Lucas, 2007; Ke & Quackenbush, 2011; Ene, Næsset & Gobakken, 2012; Hung, Bryson & Sukkarieh, 2012; Ferraz et al., 2012; Duncanson et al., 2015; Lindberg & Holmgren, 2017; Gomes, Maillard & Deng, 2018), and there have been many studies comparing delineation methods on different data types (Ke & Quackenbush, 2011; Vauhkonen et al., 2012; Eysn et al., 2015; Dalponte et al., 2015). The choice of the data to use is usually driven by data availability, but also by the characteristics of the analyzed forest. Many previous studies focus on light detection and ranging (LiDAR) data as these remote sensing data are very common in the forestry and ecology domains. LiDAR data showed to be a very powerful source of information for the detection of trees in conifer dominated forests (Vauhkonen et al., 2012), while they showed to be weaker in mature broadleaves ones. Indeed, in a mature broadleaved forest the top of the canopy is quite uniform and flat, thus the height information provided by LiDAR data is not so useful in distinguishing different tree crowns. In contrast, it could be expected that the spectral information provided by hyperspectral data could allow us to separate crowns belonging to different species. Few works exist on the use of hyperspectral data for ITC delineation (Dalponte et al., 2014, 2015), and surely this topic should be explored more in the future literature, especially given that the use of snapshots hyperspectral cameras on UAVs provide both hyperspectral information and a 3D point cloud derived using a structure from motion approach (Aasen et al., 2015).

Alignment between the delineated ITCs and the field surveyed trees is a very important step to both validate the ITC delineation results and to use the delineated ITCs in further analyses, like tree species classification and aboveground biomass prediction. It is important to verify if the ITCs that are detected on the remote sensing data are also present in the field, and also to assign the field information associated with the field trees to the ITCs in order to use them to build up prediction models of tree attributes (e.g., species and biomass prediction). The alignment procedure is usually explored in every paper dealing with ITC delineation (Eysn et al., 2015; Kandare et al., 2017). To the best of our knowledge, a standardized method to deal with this problem does not exist. This results in the use of different alignment methods for several ITC delineation paper, mostly chosen subjectively and adapted to the data used for that specific work (Ene, Næsset & Gobakken, 2012; Eysn et al., 2015; Kandare et al., 2017). In this context the idea of the NEON-NIST challenge to validate different alignment strategies on a common dataset is very important in order to arrive to a standardized method.

Tree species classification with remote sensing data is a widely covered topic in the scientific literature (Fassnacht et al., 2016). The rationale of this procedure is to use the information provided by remote sensing data to map tree species in an area. Usually this procedure is done in a supervised way, having some field data and relating them to the information provided by remote sensing data (Richards & Jia, 2006). The data used for this procedure are mainly spectral data, as different tree species are characterized by different spectral signatures. The first studies on this topic were focused on species groups, like conifer and broadleaves, as they were done using satellite multispectral data (e.g., Landsat data) that have a limited number of spectral bands and a low spatial resolution (Franklin et al., 1986; Foody & Hill, 1996). Since the 2000s, with the availability of airborne hyperspectral data, characterized by hundreds of spectral bands and a high spatial resolution, the separation of tree species become possible, and many studies focused on this topic (Fassnacht et al., 2016). Indeed, airborne hyperspectral data, due to their dense sampling of the electromagnetic spectrum, are able to characterize very small differences in the spectral signatures of trees. Moreover, the high spatial resolution, in the order of tens of centimeters, of airborne data also allows for the detection of individual trees in an image.

Here, the methods and results of team Fondazione Edmund Mach (FEM) in the NEON-NIST data science evaluation challenge are presented. The FEM team belongs to the forest ecology and bio-geochemical cycles unit of the Research and Innovation Centre of the Edmund Mach Foundation (FEM) in Italy. As presented in Marconi et al. (2018), team FEM ranked first place in Task 1 (ITC delineation) and Task 2 (data alignment), and second in Task 3 (tree species classification), thus the methods described in this paper could be considered effective methods for all the three tasks considered. The methods presented are mainly standard methods with some small adjustments to adapt to the data used. The main output of this paper and of the challenge itself is that standard methods already tested in many scenarios can lead to very good results.

## Materials and Methods

### Datasets used

The study area is the Ordway-Swisher Biological Station (OSBS) (http://ordway-swisher.ufl.edu/) that is operated by the University of Florida. OSBS comprises over 37 km^2^, has a mean elevation of 45 m.a.s.l., and is located approximately 32 km east of Gainesville in Melrose (Putnam County, FL, USA). The vegetation of the upland forests is dominated by pines and turkey oak (*Quercus laevis*) with a grass and forb groundcover. Pines are primarily longleaf pines (*Pinus palustris*) and loblolly pines (*P. taeda*). The mean canopy height is approximately 23 m.

The data, both field and remote sensing, were provided by NEON and include the following data products: (1) woody plant vegetation structure (NEON.DP1.10098); (2) spectrometer orthorectified surface directional reflectance—flightline (NEON.DP1.30008); (3) ecosystem structure (NEON.DP3.30015); and (4) high-resolution orthorectified camera imagery (NEON.DP1.30010). In greater detail, the hyperspectral data (NEON.DP1.30008) were acquired with the NEON imaging spectrometer, that is, the next generation version of the airborne visible/imaging infrared spectrometer hyperspectral imager. The data are radiometrically calibrated and characterized by 426 bands ranging from 383 to 2,512 nm with a spectral resolution of five nm. The spatial resolution is one m. The LiDAR data were acquired with the Optech Incorporated Airborne Laser Terrain Mapper Gemini. From the raw LiDAR data, a canopy height model (CHM) with one m spatial resolution was derived (NEON.DP3.30015).

**Figure 1:**
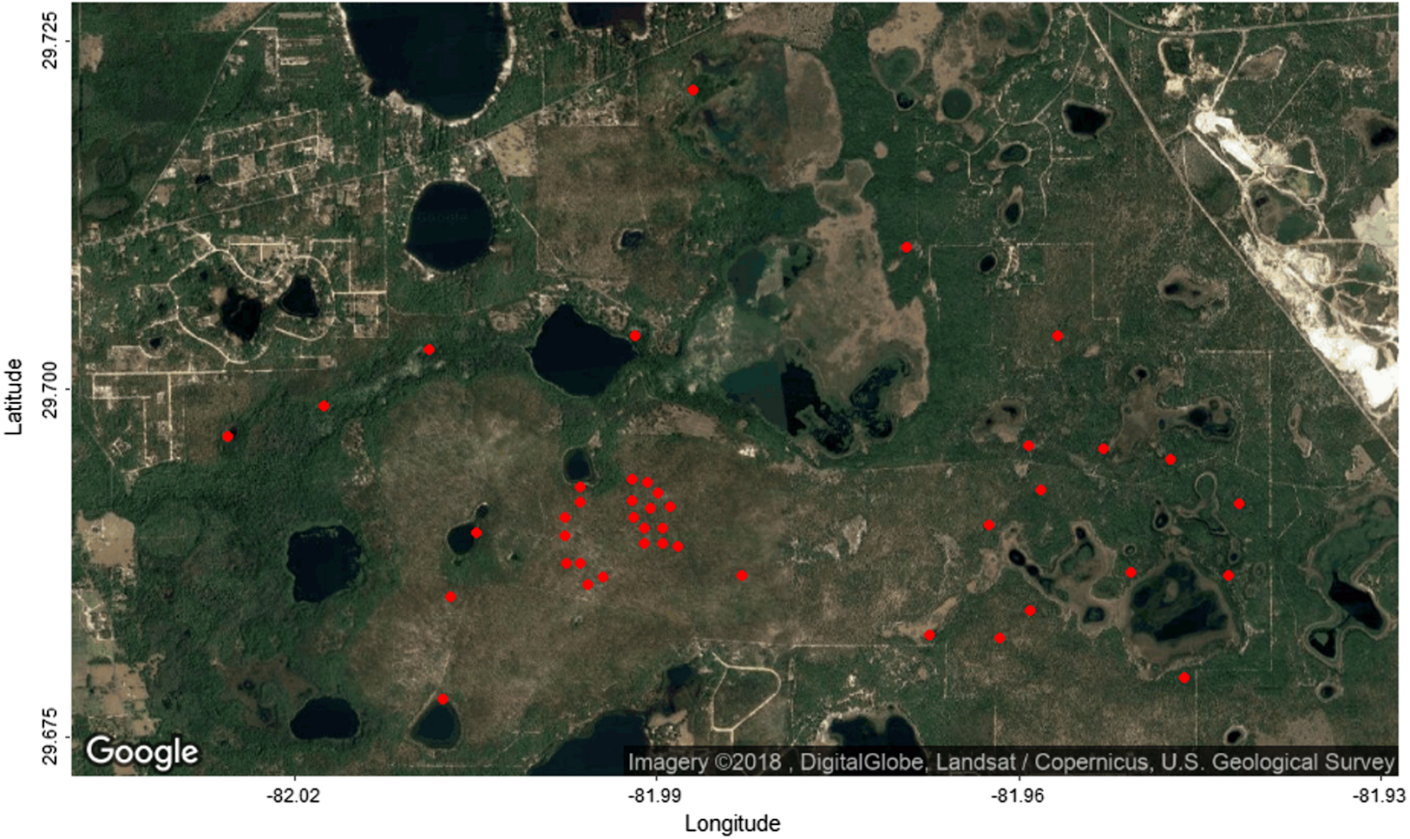
Google Maps image of the study area with the location of the 43 field plots (red dots). Image data: DigitalGlobe, Landsat/Copernicus, U.S. Geological Survey.

The following tree attributes were collected in the field: the stem ID, the location of the stem, the diameter of the stem, and for some of the trees the maximum crown radius and the radius perpendicular to the axis of the maximum radius. A total of 613 ITCs were also manually delineated in the field on airborne camera images using a tablet (Marconi et al., 2018). The field data were collected in 43 plots (see Fig. 1), among which 30 were used for training and 13 for testing. For more details see section “Task 1: ITCs delineation” and “Task 2: alignment” of Marconi et al. (2018).

For each task of the NEON-NIST challenge different datasets were used, and in particular team FEM used the following data: (i) *Task 1*: airborne hyperspectral data and the manually delineated ITCs (452 as training set and 161 as test set); (ii) *Task 2*: 89 field surveyed trees (64 as training set and 25 as test set), and the CHM data; and (iii) *Task 3*: hyperspectral data, CHM data, and 431 manually delineated ITCs for which species were recorded in the field (305 as training set and 126 as test set). The total number of species considered in Task 3 was nine: *Acer robrum* (ACRU; six training samples), *Liquidambar styraciflua* (LIST; four training samples), *P. elliottii* (PIEL; five training samples), *P. palustris* (PIPA; 197 training samples), *P. taeda* (PITA; 14 training samples), *Q. geminata* (QUGE; 12 training samples), *Q. laevis* (QULA; 54 training samples), *Q. nigra* (QUNI; five training samples), and unidentifiable species (OTHER; eight training samples).

### Task 1: ITCs delineation

The ITCs delineation was performed on the hyperspectral data using the algorithm presented in (Dalponte et al., 2015). The steps of the delineation method were:

1. The normalized difference vegetation index (NDVI) was computed for each pixel, and all pixels having NDVI below 0.6 were masked. In this way pixels belonging to non-vegetated areas were removed.
2. The hyperspectral band closest to 810 nm was selected for the delineation. The choice of this band was due to successful results obtained in previous studies (Clark, Roberts & Clark, 2005; Dalponte et al., 2014).
3. Seed points $S = \left\{ {{s_1}, \ldots, {s_N}} \right\}$ were defined using a moving window. The main idea of this method is that the pixels with the highest values of radiance (or reflectance depending on the data) are on the highest part of the trees. If the central pixel *H*(*i*, *j*) of the moving window is the pixel with the highest value of radiance, it is considered a tree top, and thus a seed:
  (1) $$H\left( {i,j} \right) \in S{\rm{\ if\ }}H\left( {i,j} \right) = {\rm{max}}\left( {{\rm{moving\ window}}} \right)$$
4. Initial regions were defined starting from the seed points. A label map *L* was defined:
  (2) $$\left\{ {\matrix{{{L_{i,j}} = k\ {\rm{if}}\ H(i,j) \in S} \hfill \cr {{L_{i,j}} = 0\ {\rm{if}}\ H(i,j)\ \notin\ S} \hfill \cr } } \right.$$
5. Starting from *L*, regions grew according to the following procedure:
  - A label map point ${L_{i,j}} \ne 0$ was considered and its neighbor pixels (NP) in the image were taken:
    (3) $${\rm{NP}} = \left\{ {H\left( {i,j - 1} \right);H\left( {i - 1,j} \right);H\left( {i,j + 1} \right);H\left( {i + 1,j} \right)} \right\}$$
  - A neighbor pixel ${\rm{NP}}\left({i',j'} \right)$ was added to the region *n* if:
    (4) $${\rm{NP}}\left( {i',j'} \right) \in \left\{ {\matrix{{{\rm{dist}}\left( {{\rm{NP}}\left( {i',j'} \right),{s_n}} \right) \lt {\rm{DistMax}}} \hfill \cr {{\rm{NP}}\left( {i',j'} \right) > \left( {{s_n}{\rm{\ *\ PercThresh}}} \right)} \hfill \cr {{L_{i',j'}} \ne 0{\rm{}}} \hfill \cr } } \right.$$
    where ${\rm{PercThresh}} \in \left({0;1} \right)$, and ${\rm{DistMax}} > 0$;
  - This procedure was iterated over all pixels that have ${L_{i,j}} \ne 0$, and was repeated until no pixels were added to any region.
6. From each region in *L* the central coordinates of each pixel were extracted, and a 2D convex hull was applied to these points.
7. The resulting polygons were the final ITCs.

The parameters of the delineation (i.e., the size of the moving window, PercThresh, and DistMax) were optimized in a supervised way using a training set made available by the organizers of the challenge: the set of parameters that provided the highest average Jaccard score (Real & Vargas, 1996) on the training set was chosen. The Jaccard score between the field ITC *A* and the delineated ITC *B* is computed as follows:

(5) $$J\left( {A,B} \right) = {{\left| {A\mathop \cap \nolimits B} \right|} \over {\left| A \right| + \left| B \right| - \left| {A\mathop \cap \nolimits B} \right|}}$$

A value equal to 1 means perfect overlap between the two delineated ITCs, while a value equal to 0 means no overlap. For each field ITCs the Jaccard score with any overlapping ITCs was computed, but only the best one was considered. The score on the training set was computed as the average of the plot level scores, which are themselves the average scores of the ITCs within each plot. The parameters used for the delineation on the test set were: a moving window size of 3 × 3 pixels, a PercThresh of 0.4, and a DistMax of 4. The implementation used is the one inside the R (R Development Core Team, 2008) package *itcSegment* (Dalponte, 2018). The results on the test set were also evaluated using the Jaccard score, and the overall confusion matrix (OCM). The OCM measures the area in square meters that is correctly or incorrectly classified as crown or not, and it accumulates the counts of area over all testing plots.

### Task 2: alignment

Alignment between the field surveyed trees and the delineated ITCs was done using a four step procedure: (1) prediction of the crown radius for the field surveyed trees for which it was not measured in the field; (2) prediction of the height for the ITCs for which this information was missing; (3) linking ITCs and field surveyed trees using an Euclidean distance based on *X* and *Y* coordinates, and height and crown radius; and (4) visual inspection of the results.

The crown radius of the field surveyed trees, for which this attribute was not measured in the field, was predicted using a relationship linking the field measured crown radius (*R*_FIELD_) with the tree height (*H*_FIELD_) and the stem diameter (*D*_FIELD_):

(6) $${R_{{\rm{FIELD}}}} = a \times {\left({{H_{{\rm{FIELD}}}} \times {D_{{\rm{FIELD}}}}} \right)^b}$$

Equation (6) was fitted using a non-linear least squares with the function *nls* of the package *stats* (R Development Core Team, 2008) of the R software.

The height of the ITCs, for which this attribute was missing, was predicted using a relationship linking the ITCs height (*H*_ITC_) and the ITCs crown radius (*R*_ITC_):

(7) $${H_{{\rm{ITC}}}} = a \times {R_{{\rm{ITC}}}}^b$$

Equation (7) was fitted using a non-linear least squares with the function *nls* of the package *stats* of the R software (R Development Core Team, 2008).

Each ITC was linked to the closest field surveyed tree according to the sum of the Euclidean distance between their position (Eq. (8)) and the Euclidean distance between their attributes (Eq. (9); height, and crown radius):

(8) $$D = {D_{{\rm{POS}}}} + {D_{{\rm{ATTR}}}}$$

(9) $${D_{{\rm{POS}}}} = \sqrt {{{\left( {{X_{{\rm{ITC}}}} - {X_{{\rm{FIELD}}}}} \right)}^2} + {{\left( {{Y_{{\rm{ITC}}}} - {Y_{{\rm{FIELD}}}}} \right)}^2}} $$

(10) $${D_{{\rm{ATTR}}}} = \sqrt {{{\left( {{H_{{\rm{ITC}}}} - {H_{{\rm{FIELD}}}}} \right)}^2} + {{\left( {{R_{{\rm{ITC}}}} - {R_{{\rm{FIELD}}}}} \right)}^2}} $$

After the linking, a visual inspection of the results on a GIS software was done to visually verify the results and to readjust some of the links.

### Task 3: tree species classification

The classification of the tree species was done with a four step procedure: (1) data normalization; (2) feature selection; (3) classification; and (4) aggregation. Data normalization was done to ensure that the pixel values were uniformly distributed across all the crowns. Each pixel value was divided by the sum of the values of that pixel in all the bands (Yu et al., 1999). In this way, the difference in radiance due to the fact that the samples are distributed on multiple images was reduced. The feature selection step is used to select the most significant features (in this case hyperspectral bands) for the considered classification problem (in this case tree species classification). A feature selection method includes a searching strategy and a separability criterion. In this study, the search strategy used was the sequential forward floating selection (SFFS) (Pudil, Novovičová & Kittler, 1994), and the separability criterion was the Jeffries Matusita distance (Bruzzone, Roli & Serpico, 1995). These methods were used successfully in previous studies (Gómez-Chova et al., 2003; Dalponte, Bruzzone & Gianelle, 2008, 2012; Dalponte et al., 2009, 2013, 2014; Dabboor et al., 2014; Padma & Sanjeevi, 2014; Tuominen & Lipping, 2016). The feature selection was applied on the hyperspectral bands of the training data using the function *varSelSFFS* in the R package *varSel* (Dalponte & Ørka, 2016). The classification was performed using a support vector machine (SVM) classifier, having as inputs the features selected at step 2 and the value of the CHM corresponding to each ITC. The SVM implemented in the R package *kernlab* (Karatzoglou et al., 2004) was used. The predicted species labels of each pixel were aggregated at crown level with a majority rule.

## Results

### Task 1: delineation

**Figure 2:**
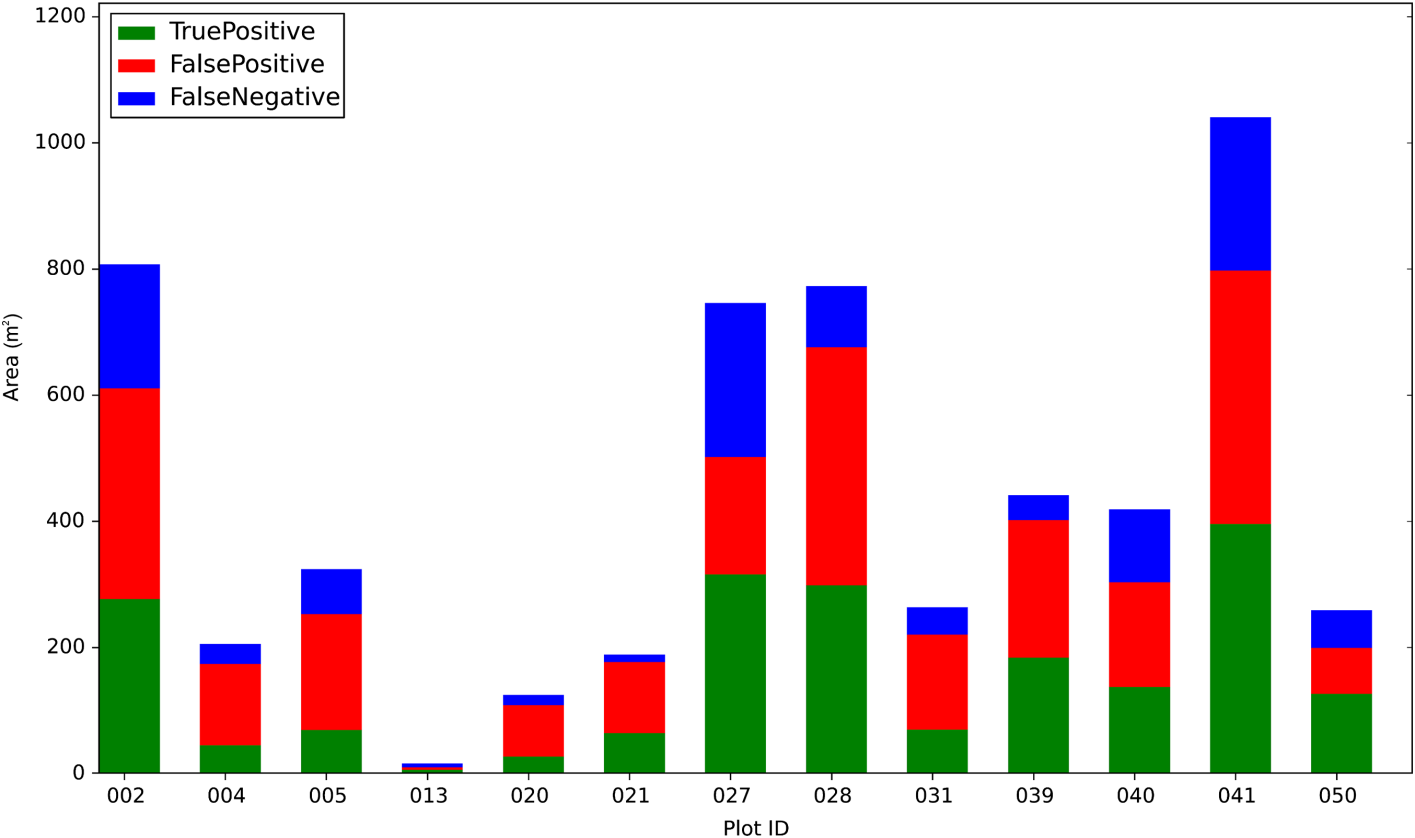
Task 1: plot level overall confusion matrix as a bar chart. *TruePositive* represent the amount of manually delineated ITCs area that was correctly delineated by the automatic delineation method used; *FalsePositive* is the amount of manually deli.

**Figure 3:**
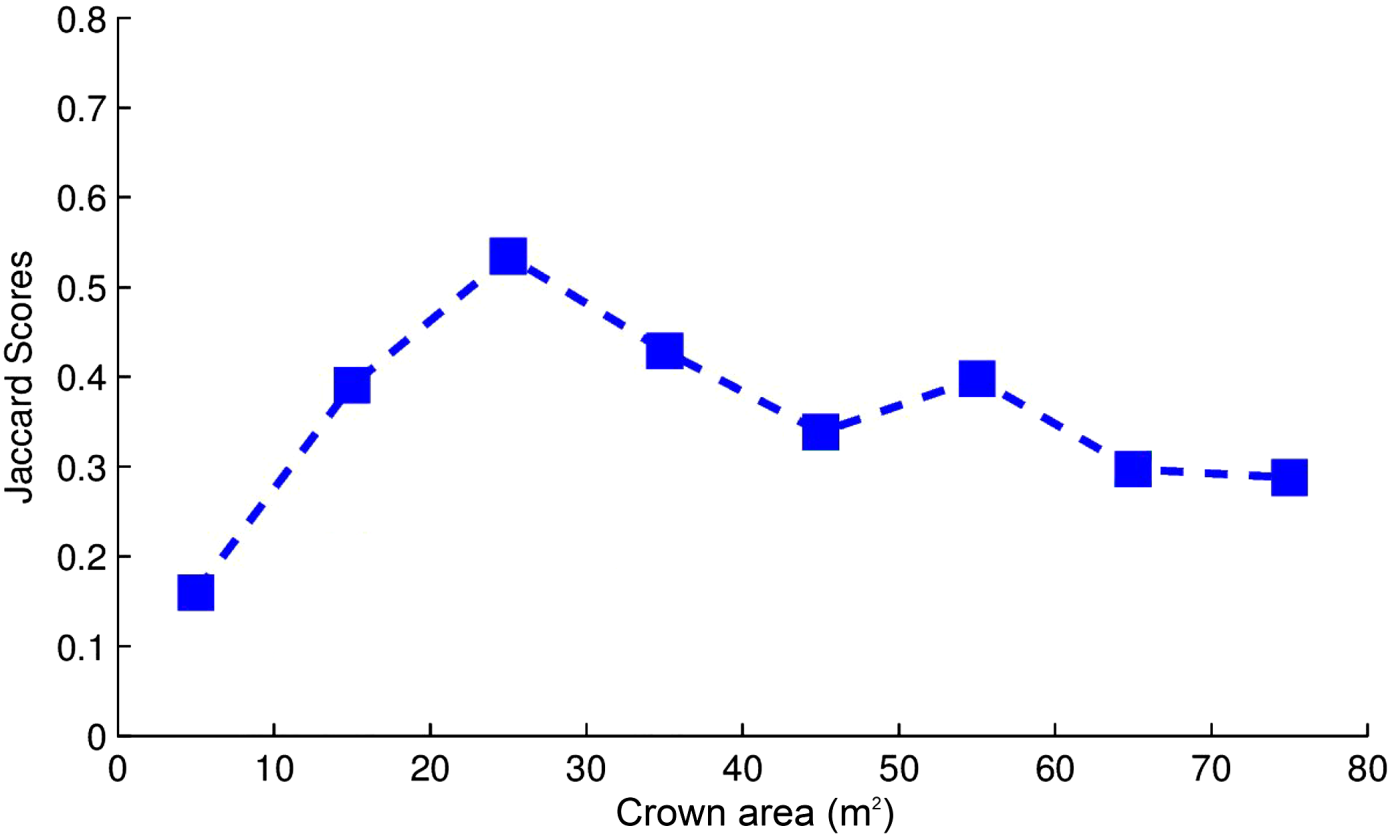
Task 1: average Jaccard score aggregated by ITCs area groups in m^2^.

The average Jaccard score for the delineated ITCs of the test set was 0.3402. This means that on average 34% of the area of the delineated ITCs overlapped the area of the field/manually delineated ITCs. Among the total area of the manually delineated ITCs (3,315.9 m^2^) 61% was correctly delineated (2,022.8 m^2^) (TruePositive in Fig. 2), while 39% was not detected (1,293.1 m^2^) (FalsePositive in Fig. 2). A total of 2,416.6 m^2^ of the delineated ITC area was wrongly delineated (FalseNegative in Fig. 2), meaning that in general all the ITCs automatically delineated were larger than the manually delineated ones. The OCM for each test plot is visualized as a bar chart in Fig. 2. The delineated area varies significantly in each plot, as well as the proportions among TruePositive, FalsePositive, and FalseNegative. The Jaccard score by crown area is shown in Fig. 3. Variability in the crown size influenced the Jaccard score, especially below 40 m^2^. The detection of small crowns (below 10 m^2^) had a lower accuracy compared to larger ones (above 40 m^2^). In particular, the proposed method showed the best results with crowns of size around 25 m^2^.

### Task 2: alignment

**Figure 4:**
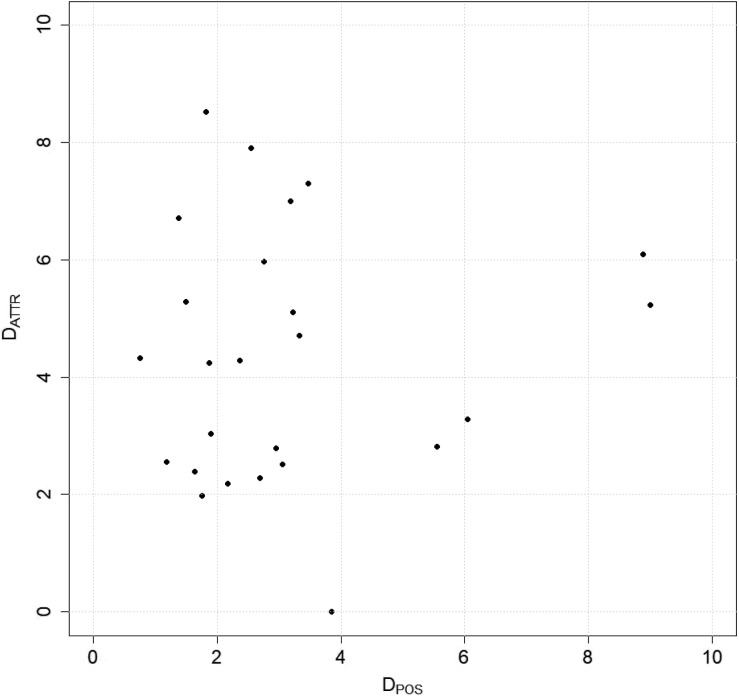
Task 2: distribution of the test set samples for the matching part according to the two components of the distance considered in the matching algorithm.

All the test ITCs were aligned with the respective field surveyed trees. In Fig. 4, the distribution of the test samples according to the two components of the Euclidean distance is shown. As it can be seen on the attributes part of the distance (*D*_ATTR_), there is high variability (between two and eight m), while for the positional part (*D*_POS_) the value is varying mainly between zero and four m.

### Task 3: tree species classification

The overall results for the considered classification task were 88.1% for overall accuracy, 75.7% for kappa accuracy, and 61.5% for mean class accuracy. From the overall performances, it is clear that the classification method used was effective, as all the performance metrics are quite good, even if the large difference existing among the overall accuracy and the mean class accuracy tells us that the classifier gave priority to the dominant species. Looking at Table 1 this is clearly visible. PIPA and QULA classes that represent the majority of the training samples have a producer’s accuracy of 91.1% and 95.5%, respectively, while classes for which the number training samples was low have a low accuracy (i.e., ACRU, PIEL).

**Table 1:** Task 3: confusion matrix of the tree species classification over the test set, and producer’s and user’s accuracies.

	ACRU	LIST	OTHER	PIEL	PIPA	PITA	QUGE	QULA	QUNI	User’s accuracy
ACRU	1	0	1	0	0	0	0	0	0	50
LIST	0	1	0	0	0	0	0	0	0	100
OTHER	1	1	0	0	0	0	1	0	0	0
PIEL	0	0	0	0	2	0	0	0	0	0
PIPA	0	0	0	1	82	0	0	1	0	97.6
PITA	0	0	0	0	4	1	1	0	0	16.7
QUGE	0	0	0	0	0	0	4	0	0	100
QULA	0	0	0	0	2	0	0	21	0	91.4
QUNI	0	0	0	0	0	0	0	0	1	100
Producer’s accuracy	50	50	0	0	91.1	100	66.7	95.5	100	

## Discussion

Team FEM ranked first for ITC delineation (Task 1) of the NEON-NIST challenge. The ITC delineation was carried out on a band of the hyperspectral images, using an approach that was already used in a previous work (Dalponte et al., 2015). The reason for this choice was that, from an initial analysis, the training ITCs provided by the organizers of the NEON-NIST challenge had a poor alignment with the LiDAR data, while they were properly aligned to the hyperspectral data. This fact was confirmed after the end of the challenge, when the organizers revealed that the training and test ITCs were manually delineated on some airborne camera images using a tablet (Marconi et al., 2018). The methods used by the other two teams participating to the challenge (teams Conor and Shawn) and the baseline method were based on LiDAR data (Marconi et al., 2018; Taylor, 2018; McMahon, 2018), which can explain their poor performances (Jaccard score of 0.184, 0.0555, and 0.0863). The comparison of the results across teams also showed that the FEM approach outperformed the other approaches in the delineation of the small trees, while it was less efficient for the large trees (Marconi et al., 2018). This is due to the fact that we decided to use a small moving window (3 × 3 pixels). Hengl (2006) suggested that in order to detect a circular object on an image (like a tree crown) it is necessary to have at least four pixels representing that object. According to this theory, in our case, using the hyperspectral images at one m resolution allowed us to detect trees with at least four m^2^ of crown size. To this constraint, it should be added that if we used a window size larger than 3 × 3 pixels, small trees could have been detected only if they were isolated from other trees. The use of a variable size moving window, like the one that is implemented for LiDAR data in the *itcSegment* library and used in (Dalponte et al., 2018), would have probably improved the final results. In a previous study (Dalponte et al., 2015), the delineation method used in the NEON-NIST competition was compared with three delineation methods based on LiDAR data. The hyperspectral delineation method outperformed the LiDAR based methods on the delineation of broadleaf tree canopies. This fact can also explain the very good performances of team FEM in the NEON NIST data science evaluation challenge because the study area had many broadleaf trees. Considering these results in the domain of ITC delineation literature it can be said that in general the results are on the average, as it is quite standard to detect 30–40% of the trees in broadleaf forests (Eysn et al., 2015). Comparing the results with others on conifer forests, especially in North Europe, the results appears quite poor, as in spruce forests the accuracy can be over 90% (Vauhkonen et al., 2012).

In the alignment task (Task 2) of the NEON-NIST challenge FEM team ranked in the first place, with all the trees correctly matched (score 1). The other participating groups, and the baseline method had a score of 0.48. The baseline prediction was the application of a naive Euclidean distance from the field tree location to the centroid of the delineated ITC. Team Conor used an approach similar to the one that we used, with a difference in the way the missing data were predicted, and the distance computed (McMahon, 2018). From the results, it seems that a great improvement was made by calculating the missing parameters. Moreover, a visual inspection of the results was essential in correctly aligning all ITC, as two trees were reassigned after this inspection. Such visual inspection is not doable over large datasets, even if, in our experience, it is always suggested as it helps in finding macroscopic errors. As mentioned in the Introduction, a correct choice of the alignment strategy depends on the type of data that can be used for this purpose. The fact that most of the works in the field of crown delineation use a different arbitrary method is not ideal. Building an alignment strategy common to everyone would simplify methods inter-comparison. In analyzing the literature, it is possible to see that the majority of methods are based either on the overlapping of the field tree positions with the delineated ITCs (Ene, Næsset & Gobakken, 2012), or on the distance both horizontal (i.e., *X* and *Y*) and vertical (e.g., height) (Eysn et al., 2015; Kandare et al., 2017). In many studies a distance constraint, both vertical and horizontal is also applied, meaning that a field surveyed tree and an ITC could be matched only if the distance is lower than a certain value (Kandare et al., 2017). This constraint is usually applied in studies where the matched information is then used for the prediction of stem attributes.

The classification task (Task 3) had the most participants and team FEM ranked in the second place. All the teams properly detected the two-dominant species (PIPA and QULA) while almost all had problems in detecting minority species (i.e., species with a low number of training samples). This is a limitation of many other methods proposed in the literature as many classifiers tend to give priority to highly represented species. Team StanfordCCB that ranked first place outperformed team FEM in the detection of PITA and OTHER species while they got the same results for the other species (Marconi et al., 2018). The fact that the first three teams in the ranking used an approach of data pre-filtering for either cleaning the training set from noise pixels or to select the most informative features, tells us that this step is quite important, and it should not be avoided. It is worth noting that this dataset is a typical example of an imbalanced dataset, where the ratio between the number of samples of the most frequent class and the number of samples of the least frequent class is very high (PIPA has 49 times samples more than LIST class). Many studies have been done on this problem, especially in the machine learning community (He & Ma, 2013; Salunkhe & Mali, 2016), and the existing studies can be classified into four groups: (i) data level approaches: these methods aim to rebalance the distribution of classes by applying resampling techniques (e.g., under-sampling or over-sampling) (Chawla et al., 2002); (ii) algorithm level approaches: these methods modify the existing algorithms in order to handle the imbalanced data (Imam, Ting & Kamruzzaman, 2006); (iii) cost sensitive learning approaches: these methods combines the data and algorithm level approaches to gain benefits of both (Peng, Chan & Fang, 2006); and (iv) classifier Ensemble techniques: these methods combine multiple diverse classifiers which disagree with each other (Salunkhe & Mali, 2016). All these approaches should be considered in the future in the ecology community as the imbalance of the datasets is quite typical in species classification where it is normal to have a forest with some dominant species and some rare ones.

## Conclusions

In this paper, the results of team FEM of the NEON NIST data science evaluation challenge were presented. The methods applied were effective as team FEM ranked first in ITC delineation task (Task 1) and the alignment task (Task 2), and second in the classification task (Task 3). The delineation method proposed was based on hyperspectral images, showing that LiDAR data are not always the best data source for ITC delineation. Importantly, pre-analysis of the available data helped significantly in the choice of the data to use. Alignment was based on both location and tree characteristics, but what probably made the big difference was the way this information was used, and the way the missing information was predicted. Indeed, another team used the same information obtaining very different results. The classification architecture adopted was quite standard, and it failed to classify rare species. As a future development, it may be interesting to combine both hyperspectral and LiDAR information in the crown delineation, and to consider classifiers especially developed for imbalanced data problems that can improve the classification of rare species.

## Additional Information and Declarations

### Data Availability

The following information was supplied regarding data availability: ECODSE group. (2017). ECODSE competition training set [Data set]. Zenodo. http://doi.org/10.5281/zenodo.867646.
